# Artificial Intelligence-Aided Headache Classification Based on a Set of Questionnaires: A Short Review

**DOI:** 10.7759/cureus.29514

**Published:** 2022-09-23

**Authors:** Bob Daripa, Scott Lucchese

**Affiliations:** 1 Internal Medicine/Neurology, Singapore General Hospital, Singapore, SGP; 2 General Physician, Grant Government Medical College and Sir J.J. Group of Hospitals, Mumbai, IND; 3 Neurology, Headache, University of Arkansas for Medical Sciences, Little Rock, USA; 4 Neurology, Headache, University of Missouri School of Medicine, Columbia, USA

**Keywords:** applications, deep learning, headache classification, machine learning, artificial intelligence

## Abstract

Wielding modern technology in the form of artificial intelligence (AI) or deep learning (DL) can utilize the best possible latest computer application in intricate decision-making and enigmatic problem-solving. It has been recommended in many fields. However, it is a long way from achieving an ambitious genuine intention when it comes to understanding and identifying any headache condition or classification, and using it error-free. No studies hitherto formalized any headache AI models to accurately classify headaches.

A machine’s job can be arduous when incorporating an emotional dimension in decision making, re-challenging its own diagnosis by keeping a differential at all times, where even experienced neurologists or headache experts sometimes find it demanding to make a precise analysis and formulate a methodical plan. This could be because of spanning clinical presentation at a given moment of time or a change in clinical pattern over time which apparently could be due to intercrossing multiple pathophysiologies.

We did a short literature review on the role of artificial intelligence and machine learning in headache classification. This brings forth a minuscule insight into the vastness of headaches and the perpetual effort and exploration headache may demand from AI when trying to scrutinize its classification. Undoubtedly, AI or DL could better be utilized in identifying the red flags of headache, as it might help our patients at home or the primary care physicians/practicing doctors/non- neurologists in their clinic to triage the headache patients if they need an imperative higher center referral to a neurologist for advanced evaluation. This outlook can limit the burden on a handful of headache specialists by minimizing the referrals to a tertiary care setting.

## Introduction and background

Headache is mostly an intricate multifarious brain manifestation to manage, despite how simple it looks. Even experienced neurologists or headache experts sometimes find it demanding to make a precise diagnosis and formulate a methodical plan. This could be because of a spanning clinical presentation apparently due to intercrossing multiple pathophysiologies. Wielding modern technology in form of artificial intelligence (AI) or deep learning (DL) can utilize the best possible latest computer application in intricate decision-making and enigmatic problem-solving. It has been recommended in many fields, but then it is a long way from achieving an ambitious genuine intention when comes to understanding and identifying any headache condition or classification, and using it error-free [1,2].

Successful utilization of AI is appreciated in law and regulation, plant disease, and medical problems [1]. For instance, its utility in hypertension diagnosis, drug discovery, and nephropathy detection among newborns is well known [3]. Furthermore, its application in cancer-associated thrombosis risk assessment and breast cancer progression risk calculation is noteworthy [4]. There are many more applications in the process of development like Alzheimer’s and Parkinson’s disease diagnosis and brain tumor classification [5].

A primary headache is a subjective phenomenon. It is challenging to integrate technology asking to make it a diagnosis considering that we do not have any objective parameters to feed into or utilize in a computerized expert system as done in the above medical situations for effortless interpretation. Constant endeavors from engineering and technology may bring high-yielding outcomes; thus, AI should be coaxed for the betterment of mankind and the healthcare system. Headache has many undeciphered dimensions. Hence, the current era demands an AI technology that can auto-upgrade and sculpt as per the new updates in this discipline.

## Review

Method

We did a literature search in Pub med and Google scholar. Title searches were “artificial intelligence (AI) in headache classification", "machine learning (ML) in headache classification" or "deep learning use in headache classification". After excluding the duplicates, only 12 studies were included that were written in the English language and met the above search criteria. Studies were from technology and technical science, neuroscience, engineering, medicine, and emergency medicine department, from 2010 to December 2021. Studies included projects, where a set of questionnaires were used, at least classified one headache type by using assorted algorithms, or where set-up models were employed in the emergency departments to codify headaches. Opinions about AI on headaches or unpublished projects were also included. As our understanding of computer programming, software, and detailed working pattern of these sophisticated models/technology was limited, we did not elaborate on the algorithmic logic, processing, and application part. 

Result 

The inference drawn from various studies from the department of technology, engineering and neurosciences conducted worldwide expressing the application of AI in headache classification are charted, summarized and illustrated well in a tabular format below (Table 1) for a better understanding. It enumerates the year and place of study, number of subjects participated, particulars of AI or ML software used, advantages and the limitations in each study.

**Table 1:** A simplified summary review of use of artificial intelligence in headache classification N: number of participants; TTH: tension-type headache; TAC: trigeminal autonomic cephalalgia; LASSO: least absolute shrinkage and selection operator; SVM-RFE: support vector machine recursive feature elimination; mRMR: minimum-redundancy maximum-relevancy; k-NN: k-nearest neighbor; TDIDT: top-down induction of decision tree; NPV: negative predictive value; LSA: latent semantic analysis; ANN: artificial neural network; NLP: natural language processing; AUC: area under curve; CDSSs: clinical decision support systems; CBR: case-based reasoning; PM: probable migraine; PTTH: probable tension-type headache; COPD: chronic obstructive pulmonary disease

Sl.No.	Author Name, Year, Place of Study	Nature of Study	Number of participants (N), Software/Application Used	Key elements/Outcome/ Learning Points	Limitations in the Study	Reference
1	Almadhoun et al [1], March 2021, Gaza, Palestine. Engineering Department	Subjects not used; Designed an expert system	Delphi programming language and clips	Diagnoses 11 headache problems	11 questions to answer, and each question has multiple sub-questions describing many symptoms. Overlapping of symptoms can cause errors in diagnosis	[1]
				Does not require any training before using this expert system	Inability to diagnose other headache types not listed in the system	
					Not checked the accuracy, specificity, or sensitivity of the expert system.	
2	Kwon et al [2], 2020 Seoul, South Korea	Retrospective study	N=2162. Divided into 2 cohorts: Training=1286, Test=876.	75 screening questions were used in details	Other less prevalent although significant primary headaches and secondary headache (other than the causes of thunderclap headaches) were excluded because of a long list of heterogenous diseases causing them.	[2]
			Stacked classifier model used with4 layers of binary XGBoost classifiers for differentiating: Migraine, (tension-type headache) TTH, (trigeminal autonomic cephalalgia) TAC, Epicranial headache, and thunderclap headache.	Stacked XGBoost classifier result: Accuracy: 81%	Used only 3 clinical features in each stack to draw insight about headache types and the clinical symptoms used here are different from (the International Classification of Headache Disorders) ICHD-3 criteria.	
			LASSO (least absolute shrinkage and selection operator) used for each stacked classifier layer. LASSO compared to SVM-RFE (support vector machine recursive feature elimination) and mRMR (minimum-redundancy maximum-relevancy).	Sensitivity for: Migraine=88%, TTH=69%, TAC=53%, Epicranial=51%, Thunderclap=51%.	The clinical course cannot be understood from these 75 questionnaires, so accurate diagnosis is difficult.	
			The selected features used XGBoost classifier which was compared to k-NN (k-nearest neighbor), SVM (support vector machine), and random forest.	Specificity for: Migraine=95%, TTH=55%, TAC=46%, Epicranial=48%, Thunderclap=51%.	Data derived from a single center.	
				Performance report in migraine classification was excellent, rest was inferior. This study can be used as pre-screening.	Conventional machine learning utilized here. No use of deep learning.	
3	Krawczyk et al [3], 2012. Wroclaw, Poland Mixed department: Technology, Technical Sciences & Medicine	Questionnaire filled by subjects where headache patients also included; ML algorithms developed and tested; a Prospective study	N=579	Best results noticed in with accuracy %: Random Forest=79.97±3.13,	Included only migraine, TTH, and loosely defined other headache types which included all remaining headaches be it primary or secondary.	[3]
			Age: 20- 65 years	Bagging=78.24±2.98,		
			Used algorithms: Naive Bayes (a probabilistic classifier), C4.5 (based on ‘Top-Down Induction of Decision Tree’ (TDIDT), Support vector Machine (SVM), Bagging (or bootstrap aggregating), Boosting, Random Forest.	Boosting 76.68±2.43		
			Used filter selection algorithms: Consistency measure filter, Relief, Genetic algorithm wrapper			
4	Julian et al [6], 2019. Study conducted in a hospital, Emergency Department. Aim: Detection of probable secondary headache	Retrospective study	N=7972. Primary headache=7098. Secondary headache=874.	Probable secondary headache: Sensitivity=89%, Specificity=73%, Negative predictive value (NPV)=98.2%.	Limited to emergency setup	[6]
			Records were processed using: Latent Semantic Analysis (LSA). Support Vector Machine (SVM) model used for training. Used Python program.	Optimized the time in the emergency	Emergent primary headaches need exploration.	
5	Messina et al. [7], April 2020. Mila, Italy. Neurosciences Department	Opinion	An opinion about Machine Learning in Headache	-	-	[7]
6	Celik et al [8], 2009.	Retrospective collecting records	Artificial immune system (computational artificial intelligence)	They were working on a headache classification project and were creating a database from the neurology department in a private hospital	Claims that would publish results after completion of the project.	[8]
					Details not known.	
7	Tezel et al. [9],	Subjects not used. Designed and developed an AI system	Clonal selection algorithm (an artificial immunity approach)	Work on headache diagnosis. Included 250 different symptoms for the training set. 150 symptoms related to headaches.	Classified into migraine headache, TTH, and set headache.	[9]
			Based on the clonal selection principle	For Test set: Correctly classified symptom set: 96.74%		
			Inspired by biological immunology.	Incorrectly classified symptom set: 3.26%		
8	Katsuki et al [10], 2020. Neurology, Neurosurgery Department. Aim: For automated primary headache diagnosis	Retrospective investigated headache database and developed a DL system	N=848	Accuracy: 0.7759	The sample size is small.	[10]
			Age: 40-74 years	Categorized into: Migraine, TTH, TAC, and Other primary headache disorders.	They did the study in a single hospital.	
			Used Deep learning framework-Prediction One.		External validation not done	
			Utilized artificial neural network (ANN) with internal cross-validation.		No separation between chronic and episodic frequent headaches of >=15 days per month to >15 days per month for migraine or TTH headache.	
			Also used Confusion matrix of model			
			Used Japanese language with onomatopoeia, therefore utilized Japanese natural language processing (NLP)			
9	Keight et al., [11]. Engineering, Medicine and Neurosurgery Department	Retrospective headache dataset collection from two medical facilities	N=836	Classified headache into Tension-Type Headache, Chronic Tension-Type Headache, Migraine with Aura, Migraine without Aura, Trigeminal Autonomic Cephalalgia.	-	[11]
			Study was done in two medical centers in Turkey.	Area under the curve (AUC): 0.985		
			9 machine learning classifiers used in a supervised learning setting	Sensitivity: 1 Specificity: 0.966		
10	Yin et al [12], 2015. China. Aim: To diagnose two headache types namely probable migraine and probable TTH	This comprehensive study worked on 3 steps viz. data acquisition through clinical interviews, construction of a case library, and lastly development of a case-based reasoning system	Clinical decision support systems (CDSSs) are based on case-based reasoning (CBR).	Can be a diagnostic tool for the general practitioner.	Inadequate case library due to complex headaches	[12]
			K-Nearest Neighbor (KNN) method implemented.	Accuracy is very high in recognizing these two headaches.	Needs multi-centric study and validation	
			N=676 cases	Earlier CBR used: (1) CASEY: to diagnose heart complication		
			Probable migraine (PM) 56.95% Probable TTH (PTTH): 43.05%	(2) Decision-based support system to diagnose (chronic obstructive pulmonary disease) COPD		
			Test set: N=222. PM: 76.1%, PTTH: 23.9%	(3) Hybrid case -based reasoning approach to diagnose breast cancer and thyroid disease.		
11	Qawasmeh et al [13], 2020. Jordan	Developed an ML-based system where its prediction accuracy checked by a web-based questionnaire’s answer	N=614 patients records. Public hospital. Males=199; Female=415. Different age group.	Hybrid model (clustering and classification): Integrated K-means clustering with Random Forest classifier	Excluded migraine with aura from this study as its differential could be a stroke.	[13]
			High-performance headache prediction support system (HPSS) was employed based on a hybrid machine learning model.	Migraine prediction accuracy=99.1%		
			Used 19 questions related to headache symptoms, according to ICHD-3 criteria.	Overall accuracy=93% (random forest)		
			26 classification algorithms were applied to 614 patients.	HPSS claimed good positive feedback from patients, medical students, and doctors. It is an easy-to-use interface that saves time and effort.		
12	Woldeamanuel et al [14], 2021. Division of Headache & Facial Pain. Stanford, CA, USA	A meta-analysis of 41 studies	Total=41 studies. Median age 43 years, 77% women. The median sample size was 288.	Used case-based reasoning, DL, classifier ensemble, ant-colony, artificial immune, random forest, white and black box combination, hybrid fuzzy expert system	60% of the digital tools were based on ICHD criteria.	[14]
			4 studies were based on a questionnaire	10 studies (25%) compared multiple ML programs	12% of tools were evaluated in non-clinical centers	
			Phone interviews in 2 studies	Diagnostic accuracy=89%, sensitivity=87%, specificity=90%	Interstudy heterogeneity of software	
			Face-to-face interview: 82% (a strong feature)		No proper patient selection method in 39% of included studies	
					No description of age or sex ratio in 25 studies	
13	Sah et al [15], 2017. Bhopal, India	Database created from headache diary and employed selection technique for analysis	Work on migraine headache classification. Used: data mining classifiers K-NN, support vector machine (SVM), Random Forest, Naïve Bays.	The best result was derived from the Naïve Bays classification. AUC 0.475, Precision 0.905	Data collected from headache diary	[15]
			18 questionnaire			
14	Liu et al [16], 2022. Shanghai, China. School of medicine	A cross-sectional study	Used ML for identifying primary headaches. This is a cross-sectional study design.	A logistic regression model was comparatively better.	Only 2 types of headaches were worked on.	[16]
			N=173 patients ( 84:migraine, 89:TTH), collected information in neurology clinics using a questionnaire (19 questions)	Logistic regression has an accuracy of 0.84 and an area under the receiver operating characteristic curve (ROC) of 0.90	Mild headache cases could not be included in this study as they did not come for medical advice.	
			Used: Decision tree, Random forest, gradient boosting algorithm, logistic regression, support vector machine (SVM) algorithms	Helped distinguish migraine and TTH and their important symptomatic distinguishing features	Small sample size	
15	Sanchez et al [17], 2020. Colombia	The study was designed to test the classifier system to distinguish types of migraine	Aimed at classifying migraine based on symptoms	ANN provided excellent results with an accuracy of 97.5% and a precision of 97%		[17]
			N=400 retrospective medical records			
			Used set of 23 variables/questionnaire of symptoms or signs			
			Implemented artificial neural network (ANN) models, logistic regression models, SVM, nearest neighbor, decision tree			
16	Celik et al [18], 2017.	A cross-sectional study to evaluate the accuracy of a classifier algorithm to diagnose primary headache type using web-based questionnaire	Aimed to diagnose primary headache based on ant colony optimization algorithm.	Classification Accuracy=96.9412%	26 patients were misdiagnosed by the ant colony classification	[18]
			The web-based questionnaire system used www.migbase.com. Used MySQL database and PHP hypertext preprocessor (PHP) programming language, 40 attributes/questions were included	Accuracies of migraine, TTH, and cluster headache were 98.2%, 92.4%, and 98.2% respectively	A similar study was done in Turkey using the same set of patients and the same website for questionnaire but implemented the artificial immune algorithms for primary headache (2015) which reached an accuracy of 99.6471% (used AIRS2-Parallel algorithm) [19]	
			N=850 headache patients from 3 cities who visited a neurologist			
			Age range= 15 to 65 years.			
			70% female and 30% male.			

 

Discussion 

Identification of focal cortical dysplasia, the evolvement of neuroimaging biomarkers in Alzheimer’s and prognosticate clinical consequences in depression therapeutics undoubtedly prove AI’s success and expanding boundaries [7]. With regards to migraine and cluster headaches, there can be a functional variation in terms of activation of separate structures in the brain, namely the trigeminovascular system, brainstem, hypothalamus and cortical areas. There could be an adjoining structural alteration of cortical thickness and its surface area. Perhaps, these changes could be related to ictal or interictal phases, and may be dynamic in nature [7]. The use of ML algorithms in functional and morphometric MRI analysis helps to distinguish these headache features [7]. It is appropriate to mention here that AI has invested a decade’s drudgery in recognizing just one headache disorder: migraine [4].

In short, numerous AI models, namely artificial neural networks (ANNs), artificial immune system (AIS) or support vector machines (SVM) are grappling to diagnose/categorize the phenotypes of only one headache type as they need further studies and validation; the exemplary model hunt is on-going. A handful of studies from the past provide little insight into AI’s influence on headache as illustrated above in table 1. and the perpetual effort that headache demands from AI when attempting to explore its own classification can be well anticipated from their limitations. Machine learning or deep learning has already been used in scouting neuroimages related to headaches [2]. In the time ahead, we anticipate AI/ML may provide neuroimage brain ‘signatures’ for specific headache categories, aided by clinical data [4].

*Challenges AI May Face Over The Classification of Headache*s * And Our Vewpoint*

The application of patient-oriented digital health gadgets can shrink the healthcare-pertinent levy as evidenced by telehealth utilization in the current COVID-19 pandemic. E-diary based headache monitoring, ML-driven products predicting headache onset or digital wearables identifying sleep time or even smartphone aided biofeed-back are a few cardinal achievements for our patients [14].

Adroit clinical interviews and reminiscence is the archetype when diagnosing primary headache which undoubtedly necessitates a holistic approach and involves neurophysiological, neuroimaging or blood biomarkers to which AI’s headache database, library and engineering part relies upon [2]. Overlapping clinical presentation at a given moment of time, change in clinical pattern over time and keeping a differential diagnosis at all times are challenges that may be confronted by AI. Imparting an emotionally delicate touch from the patient’s side of conversation and being empathetic from the physician’s end is difficult to simulate by AI technology.

It is interesting to note that one study attempted to classify emergency headache types using AI (particularly primary as well as secondary headache types). Undoubtedly, it excellently explored the secondary headache category with high sensitivity, specificity and negative predictive value while using an AI model, but meagerly addressed the primary emergent headache [6]. Optimum utilization and guidance of AI technology is not only limited to diagnosing probable migraine or probable TTH to help our general practitioner [12], but could also be extended to identify any alarming headache condition.

In our view, AI or DL could be utilized in identifying the red flags of headache, as it might help our patients at home or the primary care physicians/practicing doctors/non-neurologists in their clinic to triage the headache patients if they need an urgent higher center referral to a neurologist for advance evaluation, or whether they can continue with the current treatment in their clinic or home. This would minimize patient’s as well as doctor’s judgment errors, lessen panic situations, and finally both would feel confident when participating in a mutual discussion about symptom management. This approach can limit the burden on a handful of headache specialists by minimizing the referrals to a tertiary care setting.

## Conclusions

Supervised ML algorithms use training data which are processed and model trained to predict or classify a test data/input into predefined groups with the help of a ‘classifier’ whereas unsupervised machine learning models process input/test data via algorithm and vectors of a training set to declare result in a cluster format. Subtle changes in the brain can be picked up with AI neuroimaging for a better understanding of the disease state as mentioned earlier. However, AI/DL needs to combine anamnesis, clinical signs, and dynamic functional variation with adjoining structural imaging alteration to accurately recognize headache types. Ironically, no studies hitherto formalized any headache AI models to accurately classify headaches. 

AI needs a multidisciplinary approach from neurology, neurosurgery, neuroradiology, technology, engineering, and opinions from various other related disciplines for accelerated work that should undoubtedly be patient-centric. In the words of Gandhi, ‘The patient is the most important person in our hospital. He is not dependent on us. We are dependent on him. We are not doing him a favor by serving him. He is doing us a favor by giving us an opportunity to do so.’
